# Bier's Treatment or Unna's Paste

**Published:** 1908-04-11

**Authors:** 


### Editor's Letter-Box.

Our correspondents are reminded that prolixity is a great bar to publication, and that brevity of style and conciseness of statement greatly facilitate early insertion.

### BIER'S TREATMENT OR UNNA'S PASTE.

Dear Sir,—I was interested to read in your issue of April 4 the article on "Bier's Method and Ulcers of the Leg." The author's directions for applying the elastic bandage to the upper part of the affected limb are sufficiently clear to enable the ordinary practitioner who has not seen the method in use to avail himself henceforward of this simple therapeutic measure, not only as an auxiliary treatment for chronic ulcers, but also in a number of other conditions for which it is suitable. I quite agree that Bier's passive hyperæmia method should be tried in these tedious and unremunerative cases; because it is cheap and easy of application, and has, in my own experience and in that of others, occasionally yielded good results. But there are one or two disadvantages which at once suggest themselves to those in general practice. Thus, the method of periodic bandaging, which I believe to be much the better of the two modes, necessitates two daily visits—since the patient should most certainly remain in bed, if not throughout the day, at least whilst the hyperæmia is in progress; and no one but the medical man should ever apply the bandage. Again, there is a slight risk of serious pressure effects arising, even during an application of an hour or two only, when the doctor has left and there is no skilled attendant (such as there would be in a hospital ward or a rich woman's bedroom) to observe that things are going on as they should do. A familiar method of treating chronic non-specific ulcers of the leg, in poor old women who cannot lie up, should not be forgotten—namely, the weekly encasement of the limb in a firm but elastic splint composed of Unna's paste, separated from the floor of the ulcer itself by a disc of aseptic gauze to absorb discharges. Three applications of this admirable paste (previously melted in a gallipot), smeared generously with a brush over successive layers of carbolised gauze bandage and allowed to set until dry, provide rest, support, protection, cleanliness, and medication to the affected part. The paste can be made in bulk and kept almost indefinitely; it can be applied at the surgery or in the bedroom, and the results of patient trial are often excellent. Passive hyperæmia, whether produced by elastic bandaging or by suction, is without doubt a method of treatment of great and increasing benefit in a large number of inflammatory conditions, and every practitioner should avail himself of its possibilities. But it seems to me that its application to chronic ulcers of the leg is limited, and this principally by reason of the social status of those who are thus afflicted. On this account I stick to Unna.

Yours, etc.

April 6, 1908. X. H. P.

